# Antimicrobial and quorum sensing inhibitory activity of epiphytic bacteria isolated from the red alga *Halymenia durvillei*

**DOI:** 10.1099/acmi.0.000563.v4

**Published:** 2023-12-06

**Authors:** Mary Hannah Rose Padayao, Francis Reuben Paul Padayao, Jenny Marie Patalinghug, Gem Stephen Raña, Jonie Yee, Paul John Geraldino, Norman Quilantang

**Affiliations:** ^1^​ Applied Microbiology and Molecular Biology Laboratory, Department of Biology, University of San Carlos, Cebu City 6000, Philippines; ^2^​ Tuklas Lunas Development Center, University of San Carlos, Cebu City 6000, Philippines

**Keywords:** antimicrobial, epiphytic bacteria, *Halymenia durvillei*, quorum sensing

## Abstract

*Halymenia durvillei* is a red alga that is commonly utilized in the Philippines as food and as a source of high-value natural products for industrial applications. However, there are no studies regarding the microbial community associated with *H. durvillei* and its potential applications. This study aimed to isolate and identify the epiphytic bacteria of *H. durvillei* and determine their antimicrobial and quorum sensing inhibitory (QSI) effects. The thalli of *H. durvillei* were collected at the shores of Santa Fe, Bantayan, Cebu, Philippines. Bacterial isolates were identified using 16S rRNA, and their ethyl acetate (EtOAc) extracts were subjected to antimicrobial susceptibility tests against representative species of yeast and Gram-negative and Gram-positive bacteria. Their QSI activity against *Chromobacterium violaceum* was also determined. Fourteen distinct bacterial colonies belonging to four genera, namely *Alteromonas* (3), *Bacillus* (5), *Oceanobacillus* (1) and *Vibrio* (5), were successfully isolated and identified. All 14 bacterial isolates exhibited antibacterial effects. EPB9, identified as *Bacillus safensis *, consistently showed the strongest inhibition against *Escherichia coli *, *Staphylococcus aureus* and *Staphylococcus epidermidis *, with minimum inhibitory concentrations (MICs) ranging from 0.0625 to 1.0 mg ml^−1^. In contrast, all 14 isolates showed weak antifungal effects. Both *B. safensis* (EPB9) and *Bacillus australimaris* (EPB15) exhibited QSI effects at 100 mg ml^−1^, showing opaque zones of 3.1±0.9 and 3.8±0.4 mm, respectively. This study is the first to isolate and identify the distinct microbial epiphytic bacterial community of *H. durvillei* and its potential as an abundant resource for new antibacterial and QSI bioactives.

## Data Summary

The 16S rRNA sequences of the bacterial isolates in this study were deposited in GenBank (Table 1).

**Impact Statement**

The bacterial symbionts of many marine eukaryotes are known to produce chemically diverse metabolites with various bioactivities. This paper shows that the epiphytic bacterial community associated with *H. durvillei* is rich in antimicrobial metabolites and can potentially be used as a natural therapy against clinically significant pathogens.

## Introduction

The search for novel bioactive compounds in developing new drugs has prompted the exploration of underutilized resources in various ecosystems and environmental conditions [1]. Marine microbes, for example, have gained the attention of many researchers due to the chemical and structural diversity of the metabolites they produce and the various modes of action they exhibit [2]. Drug candidates that were purified and characterized from marine microbial symbionts showed potent bioactivities against various pathogens and diseases, including antibiotic-resistant micro-organisms, diabetes, cancer and inflammation [2–5]. However, the potential use of marine resources in developing new pharmaceuticals is still underexplored and presents a new area for natural product research.

The emergence of antimicrobial-resistant (AMR) pathogens is one of the most pressing public health concerns worldwide. Several antibiotics are no longer effective in treating these resistant strains, posing a substantial threat to millions of lives. Moreover, temperature changes due to global warming could lead to rapid increases in AMR bacteria because of increased bacterial growth rates and horizontal gene transfer [6]. Increasing temperatures lead to increased human and animal contact, facilitating the spread of pathogens that harbour new resistance mechanisms [7]. Despite the imminent threat that antimicrobial resistance poses, efforts in antibiotic research have declined in the past decades [8]. Thus, finding new antibiotics and developing new strategies for managing antimicrobial resistance is imperative.

Quorum sensing (QS) is a type of bacterial cell–cell communication that is commonly associated with the release of signals that regulate virulence and pathogenicity-related genes. Inhibition of QS has been shown to reduce the pressure to produce drug resistance in bacteria by limiting virulence and pathogenicity [9]. *Chromobacterium violaceum* is a Gram-negative bacillus that is commonly found in soil and freshwater. It is generally regarded as an opportunistic pathogen commonly affecting immunocompromised individuals [10, 11]. The production of violacein, a purple pigment produced by *C. violaceum *, is regulated through QS. Thus, the production of violacein is a valuable predictor of quorum sensing inhibitory (QSI) activity, as it is easy to visualize and quantify [12]. In addition, the production of cyanide and elastase in *C. violaceum* is heavily controlled through QS [10]. Taken together, these characteristics of *C. violaceum* allow it to be a suitable reporter strain for QSI screening.

Resazurin is a dye that changes colour from blue to pink when reduced to resorufin due to aerobic respiration of the active microbial cells. Thus, colorimetric assays utilizing the oxidation–reduction of resazurin have been widely employed in the determination of the least inhibitory concentration that could inhibit bacterial growth. This method has been found to be rapid, objective, scalable, quantitative and cost-effective, and, more importantly, it has been endorsed by the World Health Organization for the colorimetric liquid culture-based drug susceptibility assay for *Mycobacterium tuberculosis* [13–16]. The use of resazurin for minimum inhibitory concentration (MIC) determination has been successfully validated for its accuracy using the performance of standard antibiotics against several American Type Culture Collection (ATCC) strains and, in comparison, with those published by the Clinical and Laboratory Standards Institute (CSLI) [13–15, 17].

Microbial communities on the surface of macroalgae exhibit a symbiotic interaction that is crucial for the development and defence of their host alga [18]. Selvarajan *et al*. [19] reported that host-specific microbial communities are present in seaweeds, which are greatly influenced by the surface chemistry of the algal host. These epiphytic communities confer protection by producing antifouling and antimicrobials against secondary colonization of pathogenic microscopic and macroscopic epibiota, since macroalgae have no immune system [20]. This implies that marine microbes are an abundant resource of natural products that can be explored for their bioactivities.

*Halymenia durvillei* is a red alga ubiquitously distributed in the Indo-Pacific region. It is commonly known as ‘red sea lettuce’ and is characterized as having a dark red colour, branching blades with toothed margins and a firm gelatinous texture [21, 22]. In the Philippines, there are ongoing efforts to cultivate *H. durvillei* as a potential source of high-value pigments, phycoerythrin and r-phycocyanin [19]. Polysaccharides from *H. durvillei,* such as sulfated galactans, can be used in different applications in food technology, cosmetics and pharmaceutical research [23, 24]. Although the potential industrial applications of *H. durvillei* are well established, there are no studies regarding the distinct bacterial community of the algae.

This study aimed to isolate and identify the epiphytic bacterial community in the red alga, *H. durvillei*, and screen for their antimicrobial and QSI activities against different human pathogenic micro-organisms.

## Methods

### Collection and identification of *H. durvillei*

The thalli of *H. durvillei* were collected from the intertidal area at Santa Fe, Bantayan Island, Cebu, Philippines (11°09′21″N, 123°48′30″W). The collected samples were kept cool during transport and were immediately processed upon arrival at the University of San Carlos (USC) Applied Microbiology Laboratory. Morphological and molecular identification of the collected specimens was performed, and the voucher specimens were deposited at the USC Herbarium.

### Isolation of epiphytic bacteria from *H. durvillei*

The thalli of *H. durvillei* were washed with sterile filtered seawater and rinsed three times with sterile phosphate-buffered saline (PBS) to remove debris and loosely attached bacteria from the samples. The thalli were then swabbed with a sterile cotton applicator and inoculated in Zobell marine agar supplemented with nystatin (20 µg ml^−1^) (HiMedia, Mumbai, India). The cultures were incubated at 25 °C for 5 days. The colonies showing distinct morphologies were picked and streaked to isolate individual colonies [25].

### Colony morphology and biochemical characterization of bacterial isolates

The colony morphology of the bacterial isolates was noted based on form, elevation and margin. Phenotypic characterization of all 14 isolates was performed with identification as described in Bergey’s Manual of Systematic Bacteriology [26, 27]. Colony morphology for purified microbial isolates was determined using a Zeiss stereomicroscope (Carl Zeiss NTS Ltd, Germany), and the biochemical reactions were screened using analytical profile index (API) 20E strips (bioMérieux, France) for the characterization of isolates belonging to the Firmicutes group [28, 29]. Biochemical tests were conducted in accordance with the standard operating procedure per the manufacturer’s instructions. Briefly, bacterial isolates were grown on plates of marine agar overnight. A bacterial suspension was made using 5 ml of sterile saline solution after 24 h, and it was adjusted until it was comparable to the 0.5 McFarland standard with roughly 1.5×10^8^ c.f.u. ml^−1^ microbial cells. Then, 200 µl of bacterial suspension was added to each of the API strip compartments. After 24 h of incubation at 37 °C, the results were read and interpreted using the reading table provided in the manufacturer’s manual. A drop of 10 % ferric chloride (FeCl_3_) was added for the TDA test, Kovacs reagent was added for the IND test, and 40 % potassium hydroxide (KOH) (VP1) and α -naphthol (VP 2) were added for the VP test. All reactions were recorded as (+) for positive reactions and (−) for negative reactions.

### Molecular identification of bacterial isolates

Pure colonies were grown in nutrient media and harvested for genomic DNA extraction using the NucleoSpin Microbial DNA Mini kit (Macherey-Nagel, PA, USA). The 16S rRNA of each bacterial isolate was amplified by polymerase chain reaction (PCR) using the primers 27F(5’AGAGTTTGATCCGGCTCAG-3′) and 1492R(5′-GGTTACCTTGTTACGACTT-3′) [30]. The final volume of the PCR mix for each reaction was 25 µl containing TaKaRa Ex *Taq* DNA polymerase, Ex *Taq* reaction buffer (Mg2+free), magnesium chloride (MgCl2), sterile distilled water, 10 pmol of each primer and 1–10 ng of template DNA. The PCR conditions were as follows: initial denaturation at 94 °C for 2 min, 35 cycles at 94 °C for 1 min, annealing at 55–72 °C for 1.5 min, elongation at 72 °C for 1 min and final extension at 72 °C for 3 min [31]. The PCR products were electrophoresed in 1 % agarose gel and visualized under a UV transilluminator. The PCR products were sent to Macrogen (Republic of Korea) for gel extraction and DNA sequencing. The Sequencher 5.4.6 program was used to analyse the sequences gathered. The isolated bacteria were identified by comparing the consensus sequences to reference bacterial sequences using the blastn program (http://www.ncbi.nlm.nih.gov).

### Field emission scanning electron microscopy (FESEM)

The bacterial isolates that exhibited consistent antibiotic and QSI activities were further imaged using FESEM [32]. *Bacillus safensis* EPB9 and *Bacillus australimaris* EPB15 were grown in a 24-well microplate for 24 h using Zobell marine broth as the medium. The cells were allowed to attach to sterile glass coverslips submerged in the wells. Afterward, the coverslips were removed and washed twice with PBS. The samples were fixed in 2.5 % glutaraldehyde, followed by dehydration using different concentrations of ethanol (10–100 %) and critical point drying (Samdri PVT-3D, Tousimis, USA). The samples were stored in a desiccator and then coated twice with gold using a Quorum Q150R ion sputter coater (Quorum Technologies Ltd, UK). The surfaces of the bacterial samples were visualized using a Gemini Sigma 500 VP FESEM (Zeiss, USA) in high-vacuum mode at 3.0–3.5 kV.

### Preparation of crude extracts from bacterial isolates

The broth cultures of each bacterial isolate grown in 250 ml Zobell marine broth (25 °C, 7 days) were extracted with ethyl acetate (EtOAc). The organic layer was collected and concentrated to dryness using a rotary evaporator at 35 °C.

### Antimicrobial screening

The EtOAc extracts of the bacterial isolates were screened for their antimicrobial activity against Gram-positive bacteria (* Staphylococcus aureus* BIOTECH 1664 and *Staphylococcus epidermidis* BIOTECH 1800), Gram-negative bacteria (* Escherichia coli* BIOTECH 1634 and *Serratia marcescens* BIOTECH 1784) and yeast (*Candida albicans* BIOTECH 2085). All micro-organisms were acquired from the BIOTECH Philippine National Collection of Microorganisms and are not classified as MDR bacteria. Bacterial and fungal test micro-organisms were grown on Mueller–Hinton agar (HiMedia, Mumbai, India) and potato dextrose agar (HiMedia, Mumbai, India), respectively. The EtOAc extracts were dissolved in dimethyl sulfoxide (DMSO) at 100 mg ml^−1^. The antimicrobial activity of the extracts was determined using the disc diffusion method in accordance with the Clinical and Laboratory Standards Institute (CSLI) recommendation [33]. Briefly, the inoculum containing a 24 h culture of each test micro-organism, adjusted until comparable to 0.5 McFarland standard, was swabbed on agar plates. The 6 mm discs containing the test extracts were placed on top of the media and incubated for 24 h at 25 °C. The zones of inhibition for each disc were measured after that. The positive controls used for antibacterial and antifungal screening were streptomycin and fungisol, respectively. The CSLI classified the test pathogens (* S. aureus *, *S. epidermidis *, *E. coli* and *S. marcescens *) as being susceptible to the antibiotic standard streptomycin [33].

### Determination of MIC and minimum bactericidal concentration (MBC)

The MIC and MBC of the extracts were determined using the broth microdilution method with some modifications [17]. Briefly, inocula of 24 h cultures of test micro-organisms were adjusted to an absorbance value of 0.063, which is equivalent to an absorbance value of 0.5 McFarland standard (10^8^ c.f.u ml^−1^) [34], and then serially diluted to 10^5^ c.f.u ml^−1^ (OD_600_) and inoculated in a microplate. The extracts were added in concentrations of 0.0625–8 mg ml^−1^, and the cultures were incubated for 24 h at 25 °C. Afterward, 10 µl of 0.015 % resazurin was added to each well, followed by an incubation period of 4 h. The resazurin dye indicates the presence of active bacterial cells and its colour changes upon a redox reaction caused by active cell metabolism. A colour change from blue to pink after adding the dye signifies the presence of active bacteria. The MIC value per extract was assigned to the lowest concentration, where a colour change was not observed (blue). The MBC value was obtained by plating the contents of the well with the highest and lowest MIC values (blue wells) into sterile Mueller–Hinton agar. Concentrations where bacteria did not grow served as the MBC. Streptomycin was used as a positive control at 10 µg ml^−1^.

### QSI activity

The agar disc diffusion method was performed using the CLSI protocol with slight modifications to screen for the QSI activity of the crude extracts [33, 34]. *C. violaceum* NCTC 9757 was cultured in Luria–Bertani (LB) agar [1 g tryptone, 0.5 g yeast extract, 1 g sodium chloride (NaCl), 2 g peptone and 1.5 g in 100 distilled water] and incubated for 24 h at 28 °C. Standardized bacterial suspension with 1.5×10^8^ c.f.u ml^−1^ (OD_600_) was prepared. The inoculum was uniformly swabbed in LB agar plates. N-acyl homoserine lactones inducer (80 mM) was then swabbed as a second layer. Fifteen microlitres of the 100 mg ml^−1^ crude extract were loaded into sterile discs (6 mm diameter; Whatman no. 1). The prepared discs were placed on the swabbed plates aseptically. The inoculated plates were then incubated for 24 h at 28 °C. The diameters of the opaque and clear zones were then measured using a microcaliper. QSI was then calculated using the following formula:

,QSI=(r2−r1)

wherein *r*2 is the total growth and the QS inhibition zone radius and *r*1 is the clear zone radius. Cinnamaldehyde (10 mg ml^−1^) and DMSO were used as the positive and negative controls, respectively.

### Statistical analysis

IBM SPSS statistics software (trial version) (Illinois, USA) was used for statistical analysis. The nonparametric Kruskal–Wallis test was performed, followed by a pairwise comparison test adjusted by the Bonferroni correction for multiple tests.

## Results

### Isolation, biochemical characterization and molecular identification of *H. durvillei*-associated epiphytic bacteria

**Fig. 1.**
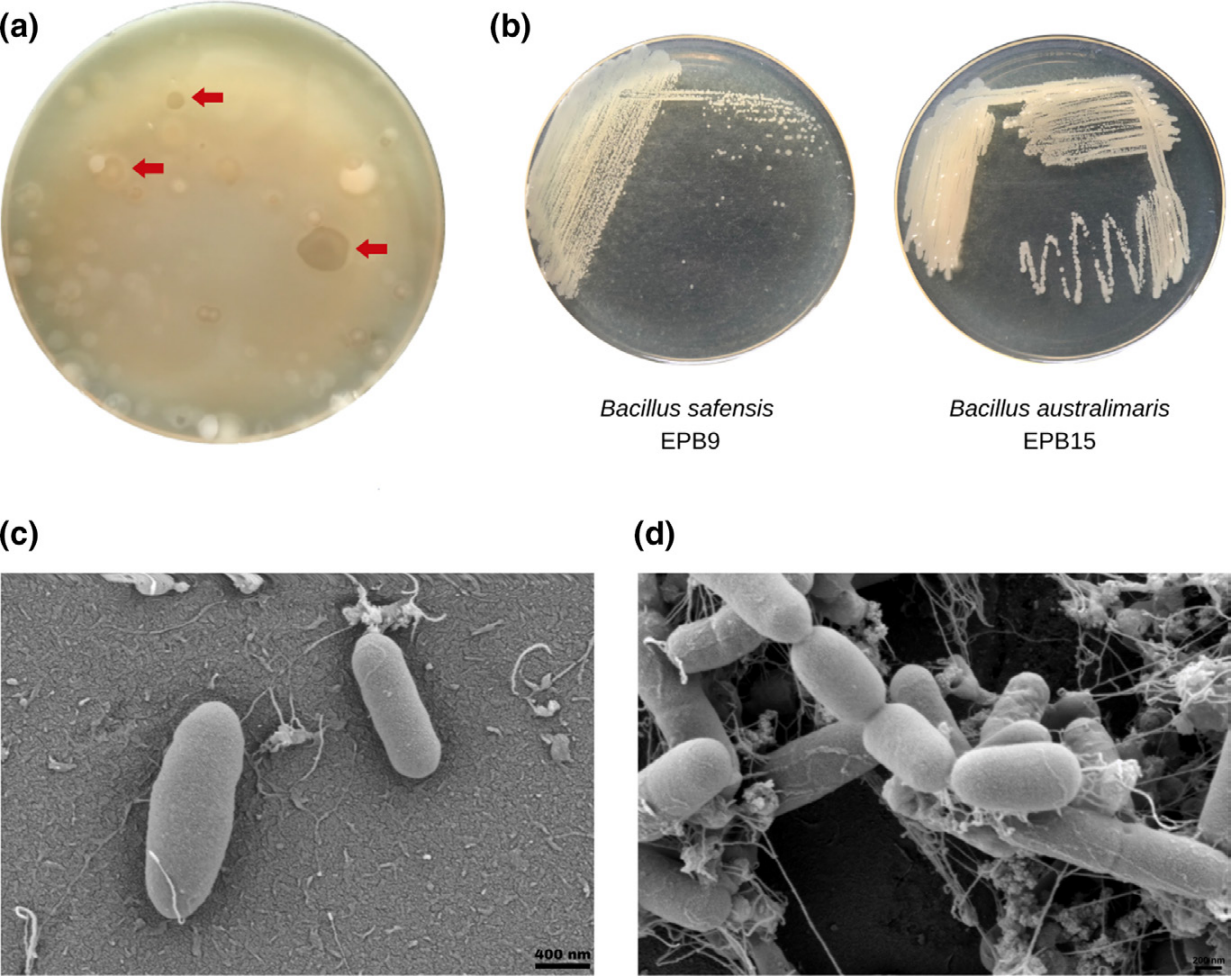
(a) Bacterial colonies obtained from the thalli of *H. durvillei* exhibited clear zones upon plating (red arrows), indicating potential production of antimicrobials. (**b**) Representative colonies of epiphytic bacteria that were successfully isolated. (**c**) FESEM photomicrographs of *B. safensis* EPB9 and *B. australimaris* EPB15.

The epiphytic bacteria associated with *H. durvillei* were isolated by swabbing the surface of its thallus and inoculating it on marine agar. Colonies that showed distinct morphologies based on form, elevation and margin were picked and purified further. Of note, several of the bacterial colonies exhibited clear zones around them, indicating some degree of antimicrobial activity (Fig. 1a). The 14 bacterial colonies that were successfully isolated were identified by comparing their consensus 16S rRNA gene sequences with reference sequences on blastn [National Center for Biotechnology Information (NCBI), http://www.ncbi.nlm.nih.gov]. Fourteen colonies were successfully identified with percentage identities >99.51 %. The identified bacteria belong to the genera *Alteromonas *, *Bacillus *, *Oceanobacillus* and *Vibrio* (Table 1). The colony morphology of 14 bacterial isolates is summarized in Table 2. The bacterial structure and morphology of two bacterial isolates showing both antibacterial and QSI activity, *B. safensis* (EPB9) and *B. australimaris* (EPB15)*,* were viewed under FESEM. *B. safensis* (EPB9) was shown as a single cell (Fig. 1c), whereas *B. australimaris* (EPB15) was seen as an aggregate of cells embedded in an extracellular matrix (Fig. 1d). In addition, biochemical characterization of bacterial isolates belonging to the Firmicutes group was performed to further determine their identity (Table 3). In particular, the results showed that both *B. safensis* (EPB9) and *B. australimaris* (EPB15) were Gram-positive and rod-shaped cells with colonies in circular form with curled margins and wrinkled elevations. This colonial morphology is in contrasts to that found by Singh *et al*. [35], who had an entire margin and raised elevation for their *B. safensi* s AS08 isolate. However, a similar biochemical profile was observed, as it was positive for catalase, oxidase, ß-galactosidase and gelatin hydrolysis. Based on morphological and biochemical characteristics, the bacterial isolates EPB9 and EPB15 were determined to be *Bacillus* species. The 16S rRNA gene sequencing further confirmed the phenotypic characterization of these two isolates as being from the phylum Firmicutes.

**Table 1.** Molecular identification of the isolated epiphytic bacteria from *H. durvillei* using 16S rRNA gene sequences

Isolate code	GenBank accession no.	Nearest related strain (GenBank accession no.)	Phylum	Query coverage (%)	Similarity* (%)
EPB1	MZ720795	* Vibrio owensii* (MN907475.1)	Proteobacteria	100	99.71
EPB3	MZ720796	* V. owensii* (CP033144.1)	Proteobacteria	100	99.64
EPB4	MZ720797	* Vibrio rotiferianus* (MT820500.1)	Proteobacteria	100	99.71
EPB5	MZ720798	* Alteromonas portus* (MG994978.1)	Proteobacteria	100	99.93
EPB7	MZ720799	* Alteromonas* sp. (KX989280.1)	Proteobacteria	100	99.85
EPB11	MZ720802	* Vibrio harveyi* (MK100328.1)	Proteobacteria	100	99.93
EPB12	MZ720803	* Vibrio alginolyticus* (MN945277.1)	Proteobacteria	100	99.86
EPB17	MZ720808	* Alteromonas* sp. (MK333154.1)	Proteobacteria	100	99.56
EPB8	MZ720800	* Bacillus* sp. (LC484680.1)	Firmicutes	100	99.79
EPB9	MZ720801	* Bacillus safensis* (MT516334.1)	Firmicutes	100	100
EPB13	MZ720804	* Bacillus altitudinis* (MT598007.1)	Firmicutes	100	99.93
EPB14	MZ720805	* Bacillus* sp. (KP119610.1)	Firmicutes	100	100
EPB15	MZ720806	* Bacillus australimaris* (MT510169.1)	Firmicutes	100	100
EPB16	MZ720807	* Oceanobacillus* sp. (MH118526.1)	Firmicutes	100	99.51

*1400–1450 base pairs were used for blast analysis.

**Table 2.** Colony morphology and Gram stain of isolated epiphytic bacteria from *H. durvillei*

Isolate code	Colony morphology				
	Form	Margin	Elevation	Gram stain	Cell shape
EPB1	Circular	Entire	Convex	Gram-negative	Rod
EPB3	Circular	Entire	Convex	Gram-negative	Rod
EPB4	Circular	Entire	Flat	Gram-negative	Rod
EPB5	Circular	Regular	Flat	Gram-negative	Rod
EPB7	Circular	Regular	Flat	Gram-negative	Rod
EPB11	Circular	Entire	Raised	Gram-negative	Rod
EPB12	Circular	Entire	Convex	Gram-negative	Rod
EPB17	Circular	Regular	Flat	Gram-negative	Rod
EPB8	Circular	Curled	Wrinkled	Gram-positive	Rod
EPB9	Circular	Curled	Wrinkled	Gram-positive	Rod
EPB13	Circular	Curled	Wrinkled	Gram-positive	Rod
EPB14	Circular	Curled	Wrinkled	Gram-positive	Rod
EPB15	Circular	Curled	Wrinkled	Gram-positive	Rod
EPB16	Circular	Contoured	Convex	Gram-positive	Rod

**Table 3.** Biochemical characterization of bacterial isolates belonging to the Firmicutes group

Biochemical test	Isolate code					
	EPB8	EPB9	EPB13	EPB14	EPB15	EPB16
Gram stain	+	+	+	+	+	+
Motility	+	+	−	−	+	−
Catalase	+	+	+	+	+	+
Oxidase	+	+	+	+	−	+
ß-galactosidase	+	+	+	+	+	+
Citrate utilization	−	−	−	−	−	−
H_2_S production	−	−	−	−	−	−
Indole production	−	−	−	−	−	−
Voges–Proskauer test	−	+	+	+	−	−
Gelatinase	+	+	+	+	+	−
NITROGEN SOURCE UTILIZATION						
Arginine decarboxylation	−	−	−	−	−	−
Lysine decarboxylation	−	−	−	−	−	−
Ornithine decarboxylation	−	−	−	−	−	−
CARBON SOURCE UTILIZATION						
Glucose	−	−	−	−	−	−
Mannitol	−	−	−	−	−	−
Inositol	−	−	−	−	−	−
Sorbitol	−	−	−	−	−	−
Rhamnose	−	−	−	−	−	−
Saccharose	−	−	−	−	−	−
Melibiose	−	−	−	−	−	−
Amygdalin	−	−	−	−	−	−
Arabinose	−	−	−	−	−	−

### Antimicrobial screening

**Fig. 2.**
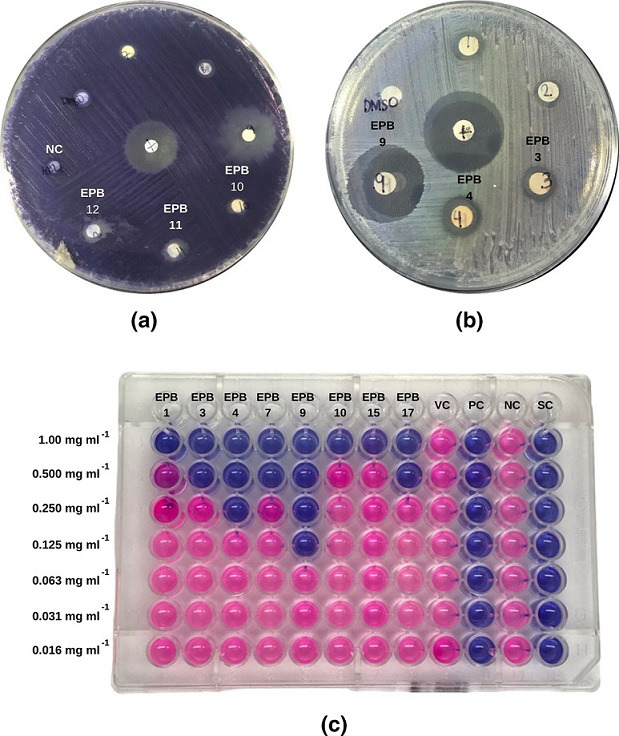
Representative plates showing the (**a**) quorum sensing inhibitory and (**b**) antibiotic activities of the bacterial isolates from *H. durvillei* against *C. violaceum* and *E. coli *, respectively. (**c**) The plate format was followed in determining the minimum inhibitory concentrations (MICs) and minimum bactericidal concentrations (MBCs) of the bacterial isolates against *E. coli *. Resazurin was added in each well after a 24 h incubation period in which blue and pink colour indicate dead and live cells, respectively. Streptomycin was used as a positive control (PC) at 10 µg ml^−1^ and DMSO as a vehicle control (VC). The negative control (NC) contains bacterial cells cultured in nutrient broth, whereas the sterility control (SC) only contains the media.

The EtOAc extracts of the identified epiphytic bacteria were screened for their antimicrobial activities against representative species of yeast (*C. albicans*), Gram-negative (* E. coli* and *S. marcescens *) and Gram-positive (* S. aureus* and *S. epidermidis *) bacteria using the agar disc diffusion method for antibiotic susceptibility testing (Table 4). The extracts of all 14 bacteria showed antibiotic effects at 100 mg ml^−1^ against Gram-negative and Gram-positive test bacteria. EPB9, identified as *B. safensis *, showed consistently strong antibiotic effects against the test bacteria (zone of inhibition >10 mm), wherein higher zones of inhibition compared with the positive control, streptomycin, were observed in *E. coli* and *S. epidermidis* (Fig. 2b). The extracts were analysed further to determine their MICs and MBCs against the test bacteria. In contrast, only three isolates (EPB11, EPB12 and EPB17) showed inhibition against *C. albicans,* with all three exhibiting weak antifungal effects compared to the positive control.

**Table 4.** Antimicrobial activity of the EtOAc extracts of the bacterial isolates from *H. durvillei* at 100 mg ml^−1^ against test micro-organisms

Isolate code	Zone of inhibition diameter (mm±sd)				
	Gram-negative bacteria		Gram-positive bacteria		Yeast
	* E. coli *	* S. marcescens *	* S. aureus *	* S. epidermidis *	*C. albicans*
EPB1	11.2±1.5^b^	7.3±1.2^a^	9.9±1.3^b^	7.7±0.5^a^	–
EPB3	11.4±2.2^b^	7.2±1.0^a^	9.8±1.4^b^	7.8±0.8^a^	–
EPB4	12.5±1.9^a^	6.8±1.0^a^	8.6±1.0^a^	9.0±1.1^a^	–
EPB5	9.6±1.4^a^	6.6±0.5^a^	7.9±1.1^a^	7.9±0.9^a^	–
EPB7	12.5±1.9^b^	6.6±0.7^a^	10.3±0.8^b^	8.2±1.0^b^	–
EPB11	10.3±1.1^a^	6.6±0.8^a^	7.6±0.8^a^	–	7.9±1.2^a^
EPB12	9.9±1.1^a^	–	7.6±0.8^a^	–	7.6±0.5^a^
EPB17	10.3±1.6^a^	7.1±0.9^a^	8.0±1.1^a^	6.6±0.7^a^	8.0±1.2^a^
EPB8	8.6±1.5^a^	7.4±0.8^a^	7.5±1.3^a^	6.4±0.8^a^	–
EPB9	21.5±2.2^b^	8.6±2.1^a^	17.0±1.9^b^	15.9±1.9^b^	–
EPB13	9.8±0.4^a^	8.2±1.6^b^	7.6±1.3^a^	9.2±1.8^b^	–
EPB14	8.8±1.1^a^	6.2±0.4^a^	7.1±1.0^a^	7.4±0.5^a^	–
EPB15	10.1±0.8^a^	7.8±0.4^b^	6.9±1.1^a^	7.1±0.7^a^	–
EPB16	8.9±1.2^a^	6.5±0.7^a^	7.1±1.5^a^	6.6±0.7^a^	–
Streptomycin	18.9±0.7^b^	15.4±1.7^b^	20.4±2.1^b^	12.4±1.0^b^	–
Fungisol	–	–	–	–	22.3±1.2^a^

Zone of inhibition, including the diameter of the filter paper disc (6 mm); mean value of three independent experiments±standard deviation.

^a, b^The different letter superscripts indicate significant difference (*P*<0.05) and groups that do not share the same letter are significantly different from each other.

Streptomycin was used as a positive control against the test bacteria at 10 µg ml^−1^.

Fungisol was used as a positive control against *C. albicans.*

–, no activity.

### MIC and MBC of the isolated bacteria from *H. durvillei*

The MICs and MBCs of the EtOAc extracts of the bacterial isolates that showed consistently strong antimicrobial activities against the test bacteria were determined (Table 5). The colorimetric reaction observed in each well after the addition of resazurin was used to determine the MIC and MBC of each extract (Fig. 2c). EPB9 showed a very low MIC for *E. coli* (0.0625 mg ml^−1^), *S. aureus* (1 mg ml^−1^) and *S. epidermidis* (1 mg ml^−1^). The rest of the extracts exhibited MICs against the test bacteria ranging from 0.25–8 mg ml^−1^.

**Table 5.** Minimum inhibitory concentrations (MICs) and minimum bactericidal concentrations (MBCs) of the isolated epiphytic bacteria from *H. durvillei* against test bacteria

Isolate code	* E. coli *		* S. marcescens *		* S. aureus *		* S. epidermidis *	
	MIC	MBC	MIC	MBC	MIC	MBC	MIC	MBC
EPB1	0.5	1	>8	>8	2	4	8	>8
EPB3	0.25	0.5	>8	>8	2	4	2	4
EPB4	0.125	0.25	>8	>8	1	2	2	4
EPB7	0.25	0.5	>8	>8	2	4	4	8
EPB17	0.25	0.5	>8	>8	2	4	8	>8
EPB9	0.0625	0.125	>8	>8	1	2	1	2

MICs and MBCs are expressed in mg ml^−1^.

### QSI activity

The QSI effects of the EtOAc extracts of the bacterial isolates against *C. violaceum* were determined using the agar disc diffusion method (Fig. 2a). *B. safensis* EPB9 and *B. australimaris* EPB15 exhibited QSI effects at 100 mg ml^−1^. Both extracts showed higher QSI activity than the positive control, cinnamaldehyde (Table 6). The rest of the extracts did not exhibit any QSI activity.

**Table 6.** Inhibition and opaque zones of EtOAc extracts of bacterial isolates from *H. durvillei* against *C. violaceum* at 100 mg ml^−1^

Isolate code	Clear zone (mm±sd)	Opaque zone* (mm±sd)	Anti-QS (mm±sd)
EPB1	7.0±0.8	7.0±0.8	0
EPB3	7.3±1.1	7.3±1.1	0
EPB4	7.2±0.9	7.2±0.9	0
EPB5	8.8±1.1	8.8±1.1	0
EPB7	6.0±0.9	6.0±0.3	0
EPB11	6.7±0.9	6.7±0.9	0
EPB12	6.2±0.6	6.2±0.6	0
EPB17	6.7±0.9	6.7±0.9	0
EPB8	6.6±0.7	6.6±0.7	0
EPB9	13±1.5	16.1±2.0	3.1±1.0^a^
EPB13	6.0±0.0	6.0±0.6	0
EPB14	6.4±0.8	6.4±0.8	0
EPB15	12.2±2.0	16.0±2.6	3.8±1.8^a^
EPB16	6.0±0.0	6.0±0.0	0
Cinnamaldehyde	21.2±2.3	23±2.4	1.8±0.4^a^

Zone of inhibition, including the diameter of the filter paper disc (6 mm); mean value of three independent experiments±standard deviation.

^a^The different letter superscripts indicate significant difference (*P*<0.05) and groups that do not share the same letter are significantly different from each other.

*Area showing visible bacterial growth without the purple coloration.

Cinnamaldehyde was used as a positive control at 10 mg ml^−1^.

## Discussion

The host seaweed and its microbiota are in such a close association that they act like a single unified organism, otherwise known as a holobiont [18]. Evidence suggests that seaweed microbiota is host-specific and greatly influenced by algal metabolites and surface architecture [36]. Several studies examining the microbial epibiota of different marine seaweeds have shown that the major phyla on their surfaces are Proteobacteria, Firmicutes and Bacteriodetes [18, 37]. Moreover, these microbes are predominantly Gram-negative and exhibit motility, indicating that they form biofilms. Motile bacteria respond to stressful situations using negative chemotaxis (e.g. swarming away), resulting in the transition from the unicellular and planktonic stage to the biofilm stage of bacterial growth and loss of flagellar motility. The activation of negative chemotaxis acts as a catalyst for the development of biofilms and other defence mechanisms against unfavourable environmental conditions [38, 39]. The signalling molecule c-di-GMP in Gram-negative bacteria inhibits flagellar motility and encourages the production of adhesion factors and extracellular polymeric substances (EPSs) for biofilm formation [40]. A study reported that the 31 phylotypes of epiphytic bacteria associated with *H. floresii* are primarily composed of groups from Proteobacteria (84 %), Bacteriodetes (13 %) and Firmicutes (3 %) [41]. Consistent with these studies, our results show that most of the bacterial isolates from *H. durvillei* are Gram-negative and are members of the group Proteobacteria (57 %), except for six species belonging to Firmicutes (43 %) (Table 1). The members of the group Bacteriodetes were not identified in this study. In addition, the bacterial isolates from *H. durvillei* belong to four genera: *Alteromonas *, *Bacillus *, *Oceanobacillus* and *Vibrio *. Among them, members of the genera *Vibrio* and *Alteromonas* are reported to cause diseases in marine eukaryotes such as fishes, crustaceans, corals and seaweeds [42, 43]. Interestingly, these species of bacteria were isolated from the healthy thalli of *H. durvillei*. Studies regarding the interactions between these bacterial isolates and their host alga can be further explored to elucidate their pathogenicity. In contrast, there are no reports regarding the pathogenicity of *Bacillus altitudinis *, *B. australimaris *, *B. safensis* and *Oceanobacillus* to marine organisms. Furthermore, this study only identified bacterial species that could grow in laboratory conditions. It did not consider all the micro-organisms present in *H. durvillei*. To achieve a more comprehensive examination of the microbial flora of *H. durvillei*, a metagenomics approach is needed.

The epibiota of marine seaweeds are reported to deter the attachment of other surface colonizers by producing antimicrobials and antifouling metabolites [36]. Several studies have shown that extracts from the epiphytic bacteria of various seaweed species exhibit potent *in vitro* antimicrobial activity against a wide range of human pathogenic bacteria [44, 45]. All 14 bacterial isolates from *H. durvillei* exhibited antibiotic effects against representative Gram-negative and Gram-positive bacteria species. In particular, *V. owensii* (EPB1), *V. owensii* (EPB3), *V. rotiferianus* (EPB4), *Alteromonas* sp. (EPB7), *Alteromonas* sp. (EPB17) and *B. safensis* (EPB9) displayed the strongest and most consistent activity among the bacterial isolates. *V. owensii* and *V. rotiferianus* are well-documented pathogens of various marine crustaceans. However, several studies have reported that certain strains of *V. owensii* and *V. rotiferianus* exhibit antibiotic activity against a range of human pathogens. This activity is attributed to the production of secondary metabolites, including carotenoid pigments and furan derivatives [46–48]. *Alteromonas* spp. are commonly known for their role in the degradation of algal polysaccharides, which is a crucial aspect of marine carbon cycling [49]. Furthermore, *Alteromonas* spp. have been shown to produce antifouling and antimicrobial compounds, such as hydrolyzing enzymes, questiomycins and polysaccharides [50–52]. Lastly, isolate EPB9, identified as *B. safensis,* exhibited the most potent inhibitory effects against the test bacteria.

*B. safensis* was first identified as a contaminant from spacecraft and on the surfaces of assembly facilities [53]. Since then, *B. safensis* has been isolated from habitats that were relatively extreme for other micro-organisms to grow in. Such habitats include rhizospheres of plants, feather dumping sites, sediments from offshore oilfields, olive oil-contaminated soil, saline desert soil and explosive-contaminated soil [54–57]. Different strains of *B. safensis* have been shown to produce various enzymes that are important in many industrial applications, such as inulinases [57], proteases [56], keratinases [55], xylanases and pectinases [54]. In addition, the presence of oxidoreductases in *B. safensis* and its ability to resist various metals such as Al, As(V), As (III) and Cr (VI) have been reported, which makes *B. safensis* a good candidate for bioremediation and biodegradation studies [58, 59]. Moreover, the antimicrobial activity of *B. safensis* has been reported in the literature. Surfactin, a biosurfactant extracted from *B. safensis* F4, was reported to have potent antimicrobial activity against Gram-positive bacteria such as *B. subtilis* and *S. epidermidis *. It also had moderate activity against Gram-negative bacteria such as *E. coli* [54]. Crude extracts of *B. safensis* OPL 19, an endophyte of *Ophioglossum reticulatum L*., were also reported to have antimicrobial activity [60]. The chitinase enzyme of *B. safensis* exhibits antifungal activity against the pathogenic *Macrophomina phaseolina* and *Rhizoctonia solani* by dissolving the cell wall component of the fungal cells [61].

Quorum sensing (QS) is a mechanism used for intra- and interspecific communication by most bacteria. This process is mediated by the production of autoinducers, which are signalling molecules that accumulate as cell density increases and, upon reaching a threshold, trigger a cascade of signal transductions that regulate many critical bacterial functions, such as sporulation, bioluminescence, virulence, reproduction and biofilm formation [62]. QS is a commonly observed process in marine epibiotic relationships modulating the settlement of microbial communities on the surfaces of the host individual [63]. Marine seaweeds produce metabolites that have been shown to inhibit, stimulate and inactivate QS signals in bacteria [64–66]. Similarly, seaweed-associated bacteria utilize QS signalling molecules to prevent the settlement of other epibionts and influence the settlement of algal zoospores [36]. Accordingly, marine epiphytic bacteria present a potentially rich source of novel bioactive compounds that can be explored for drug discovery and development. In this study, *C. violaceum* NCTC 9757 was used as a reporter strain to screen for the anti-quorum sensing activity of the bacterial isolates from *H. durvillei* [10]. The expression of the purple pigment, violacein, produced by *C. violaceum* is regulated by QS. Thus, the inhibition of violacein production is used in the present study for the preliminary screening of QSI effects.

Our results showed that two bacterial isolates from *H. durvillei*, *B. safensis* EPB9 and *B. australimaris* EPB15 exhibited QSI activities against *C. violaceum *. Gram-positive bacteria, such as members of Bacillales, utilize peptides as autoinducers for QS, whereas Gram-negative bacteria, such as *C. violaceum,* use acyl-homoserine lactones (AHLs). Differences in QS molecules signify the presence of other species, and the bacteria can act on it either by translating antimicrobial compounds or by inviting them in a biofilm. QSIs can target signal detection and signalling molecules. The QSIs produced from micro-organisms affect QS of bacterial pathogens through suppression of AHL synthesis, degradation of AHL molecules and competitive binding to receptor proteins [10]. Anti-virulence drugs, often known as QSIs, target SAM analogues, enzymes and antagonists in Gram-negative bacteria to block AHL formation, inactivate AHL enzymes and prevent AHL production, whereas for Gram-positive bacteria, they target apolipoprotein B to inactivate AIPs and block membrane-associated receptors [10, 11, 58, 59]. It is reported that some Firmicutes, such as *Arthrobacter *, *Bacillus* and *Oceanobacillus *, produce quorum-quenching enzymes that degrade AHLs and *Bacillus* sp. containing AHL lactonase that breaks down AHL signalling molecules [11]. Further studies are still needed to understand the mechanisms behind the inhibition using bacterial crude extracts of *B. safensis* and *B. australimaris. B. safensis* isolated from soil has been reported to inhibit melanin production from *Cryptococcus neoformans* and *Cryptococcus gattii*. Melanin pigments are released by cryptococcal cells through QS activity, which contributes directly to immune-modulatory activity during human infection [29]. *B. australimaris* has been shown to have antifungal activity against *Magnaporthe orayzae* [67]. However, QS activity of *B. australimaris* has not yet been reported.

There are limited studies regarding the role of *B. safensis* as a macroalgal symbiont, but it has been shown to be a marine sponge symbiont. Thus, this study is the first to identify *B. safensis* as a distinct symbiont of *H. durvillei*. In addition, this study was the first to report the isolation and identification of *B. safensis* in the Philippines. However, *B. safensis* has been isolated from both marine and terrestrial sources from neighbouring countries such as Thailand, Taiwan, the Republic of Korea and PR China. In these studies, *B. safensis* was either identified as a eukaryotic symbiont or was isolated from the environment directly [68–71].

The results of this study provide baseline information regarding the epiphytic bacterial community associated with *H. durvillei* and their antimicrobial and QSI activities. In particular, *B. safensis* EPB9, one of the bacterial isolates from *H. durvillei*, has shown promising antibacterial properties and QSI effects. These microbial symbionts can be further explored for their various applications in medicine and drug development. The isolation and identification of the bioactive compounds present in these bacterial isolates will reveal the underlying mechanisms behind their bioactivity. A metagenomics approach in identifying bacterial communities will further deepen our understanding of the complex relationship between *H. durvillei* and its associated microbiota.
